# Wide-field OCT-angiography assessment of choroidal thickness and choriocapillaris in eyes with central serous chorioretinopathy

**DOI:** 10.3389/fphys.2022.1008038

**Published:** 2022-10-21

**Authors:** Yang Meng, Yishuang Xu, Lu Li, Yu Su, Lu Zhang, Changzheng Chen, Zuohuizi Yi

**Affiliations:** ^1^ Department of Ophthalmology, Renmin Hospital of Wuhan University, Wuhan, China; ^2^ Department of Ophthalmology, The Central Hospital of Wuhan, Wuhan, China

**Keywords:** wide-field imaging, OCT-angiography, central serous chorioretinopathy, choroid, choroidal thickness, choriocapillaris

## Abstract

**Purpose:** To assess wide-field changes in choroidal thickness and choriocapillaris in eyes with central serous chorioretinopathy (CSC) compared with the fellow eyes and eyes from healthy individuals using wide-field swept-source (SS) OCT-Angiography (OCTA).

**Methods:** A cross-sectional study in which 68 eyes from 34 individual patients affected by unilateral CSC and 32 eyes of 32 age- and sex-matched healthy subjects were evaluated. All subjects underwent wide-field SS-OCTA examination to quantify choroidal thickness and vascular density of the choriocapillaris. To assess the wide-field changes, we developed five 4-by-4 mm square regions located in the posterior pole and in the four quadrants of the peripheral retina (superotemporal, inferotemporal, superonasal, and inferonasal subfields, respectively).

**Results:** The choroidal thickness of eyes with CSC was greater than that of the fellow eyes in the central and inferonasal subfields (*p* < 0.001 for the central subfield and *p* = 0.006 for the inferonasal subfield, respectively). Compared with the choroidal thickness of healthy eyes, that of patients with CSC were significantly greater in all the subfields (*p* < 0.05 for the fellow eyes and *p* < 0.05 for eyes with CSC, respectively). Compared with that of healthy eyes, the vascular density of choriocapillaris in eyes of patients with CSC were significantly greater in the central and superotemporal subfields (*p* < 0.05 for the fellow eyes and *p* < 0.05 for eyes with CSC, respectively). In the central region, the vascular density of choriocapillaris of the fellow eyes was greater than eyes with CSC (*p* = 0.023).

**Conclusion:** CSC appears to be a bilateral disease with asymmetric manifestations. Local factors of the diseased eyes may play an important role in the development of CSC, during which dynamic and regional changes in the choriocapillaris may have happened. Wide-field swept-source OCTA provided a useful tool to study the pathogenesis of CSC.

## Introduction

Central serous chorioretinopathy (CSC) is a vision-threatening chorioretinal disease characterized by localized serous detachment of the neurosensory retina (Daruich et al., 2015; van Rijssen et al., 2019; Xu et al., 2021). CSC is primarily seen in young to middle-aged male individuals and has been estimated to be the fourth most common non-surgical retinopathy (Liew et al., 2013; Daruich et al., 2015; Ishikura et al., 2022). Since 2014, CSC has been recognized as a form of pachychoroid disease, primarily defined by an abnormal increase of choroidal thickness with accompanying dilatation of the large choroidal vessels (Gallego-Pinazo et al., 2014). While there are a wide variety of studies on CSC, the underlying pathophysiological mechanisms of CSC are not yet fully understood. The most current thinking assumes that CSC is a major kind of venous overload choroidopathy, and the vision loss caused by CSC is due to venous decompensation in the choroid (Spaide et al., 2022).

Technological advances in OCT and OCT-Angiography (OCTA) have greatly improved our ability to study the choroidal changes in eyes with CSC. Spectral-domain (SD) OCT with enhanced depth imaging (EDI) and high-penetrating swept-source (SS) OCT have revealed changes in the structure of the retina, choroid, and even the sclera in eyes with CSC (Chung et al., 2018; Han et al., 2020; Imanaga et al., 2021). In short, eyes with CSC have thinner outer retinal layer thickness, thicker subfoveal choroid thickness with dilated veins in the Haller layer, and thicker scleral thickness (Chung et al., 2018; Han et al., 2020; Imanaga et al., 2021). However, these choroidal changes have only been observed in the macular region as the conventional OCT devices used in previous studies have a limited scanning area. Therefore, the changes in the choroid in the peripheral regions are rarely investigated in published studies.

Wide-field (WF) OCT has recently been used to assess peripheral choroidal changes in eyes with CSC, but studies to date have drawn inconsistent conclusions (Ishikura et al., 2022; Izumi et al., 2022). For example, in a study, Ishikura et al. (2022) found that the choroidal thickness in CSC eyes was significantly greater in all peripheral subfields compared with that in normal eyes, while in another study Izumi et al. (2022) observed no difference at the periphery. As such, CSC-induced changes in the choroidal thickness in the peripheral regions remain controversial. Moreover, there is yet no study to evaluate the peripheral vascular density (VD) of the choriocapillaris (CC) in eyes with CSC.

A recently developed wide-field SS-OCTA system has an A-scan rate of 400 kHz and a central wavelength of 1,060 nm, delivering a shorter acquisition time and deeper scan depth (TowardPi BMizar). Furthermore, the system can collect data from an area of up to 20 mm in height and 24 mm in width (∼120° angular field of fundus view) in a single scan. This allows us to explore choroidal changes across a more peripheral area than is possible with traditional OCTA devices (Xuan et al., 2022).

Therefore, the aim of this study is to investigate the wide-field changes in choroidal thickness and choriocapillaris in the diseased and fellow eyes in patients with CSC using wide-field swept-source OCT-Angiography (WF SS-OCTA).

## Materials and methods

This cross-sectional study was approved by the Ethical Review Committee of the Renmin Hospital of Wuhan University. The research was conducted in accordance with the Declaration of Helsinki and all participants provided informed consent prior to inclusion in the study.

### Patients

This study was conducted from January 2022 to March 2022 at Renmin Hospital of Wuhan University, Wuhan, China. We performed detailed ophthalmic examinations on each subject, including slit-lamp biomicroscopy, best-corrected visual acuity, intraocular pressure, fundus fluorescein angiography (FFA), indocyanine green angiography (ICGA), SS-OCT, and SS-OCTA. Diagnosis of CSC was based primarily on the angiography results, specifically, if the patient exhibited focal dye leakage from the retinal pigment epithelium on FFA, and choroidal vascular hyperpermeability on ICGA. For CSC sufferings, we assessed both the diseased eyes (treatment-naïve unilateral CSC) and the fellow eyes.

### Inclusion and exclusion criteria

To be included, subjects had to have: 1) no indication of CSC in the fellow eye; 2) intraocular pressure (IOP) of 10–21 mmHg in each eye; and 3) been afflicted for ≤9 months. Subjects were excluded if they had: 1) a history of trauma or ocular surgery other than cataract surgery; 2) any severe systemic disease; 3) any other chorioretinal diseases such as choroidal neovascularization, polypoidal choroidal vasculopathy, or uveitis; 4) were pregnant; 5) had used glucocorticoids within the last 12 months; 6) spherical equivalent <−5D or 7) consumed coffee or tea within 1 day of the OCTA examination.

### Wide-field swept-source OCT-angiography assessment of eyes with central serous chorioretinopathy

The wide-field swept-source OCT-Angiography device (TowardPi BMizar, TowardPi Medical Technology, Beijing, China) uses a swept laser source with a wavelength centered at 1,060 nm and a scan rate of 400,000 A-scans per second. A bandwidth of 100 nm enables an axial optical resolution of 3.8 μm and a transverse resolution of 10 μm. For the 24 mm × 20 mm scanning protocol we used, a single acquisition produces a WF SS-OCTA scan image is 24 mm in width (1536 B scans), 20 mm in height (1,280 pixels), and 6.0 mm in depth. The field of view is approximately 120° for a 24 mm × 20 mm OCTA image. No additional lenses or device modifications were needed during the acquisition. All OCTA scans were centered on the foeva and performed without rotation. The segmentation was performed automatically using built-in software. If necessary, manual adjustment on the segmentation was conducted to fix segmentation errors. All OCTA images were obtained by an experienced operator (YM) and reviewed by another two authors (ZY and CC).

**FIGURE 1:**
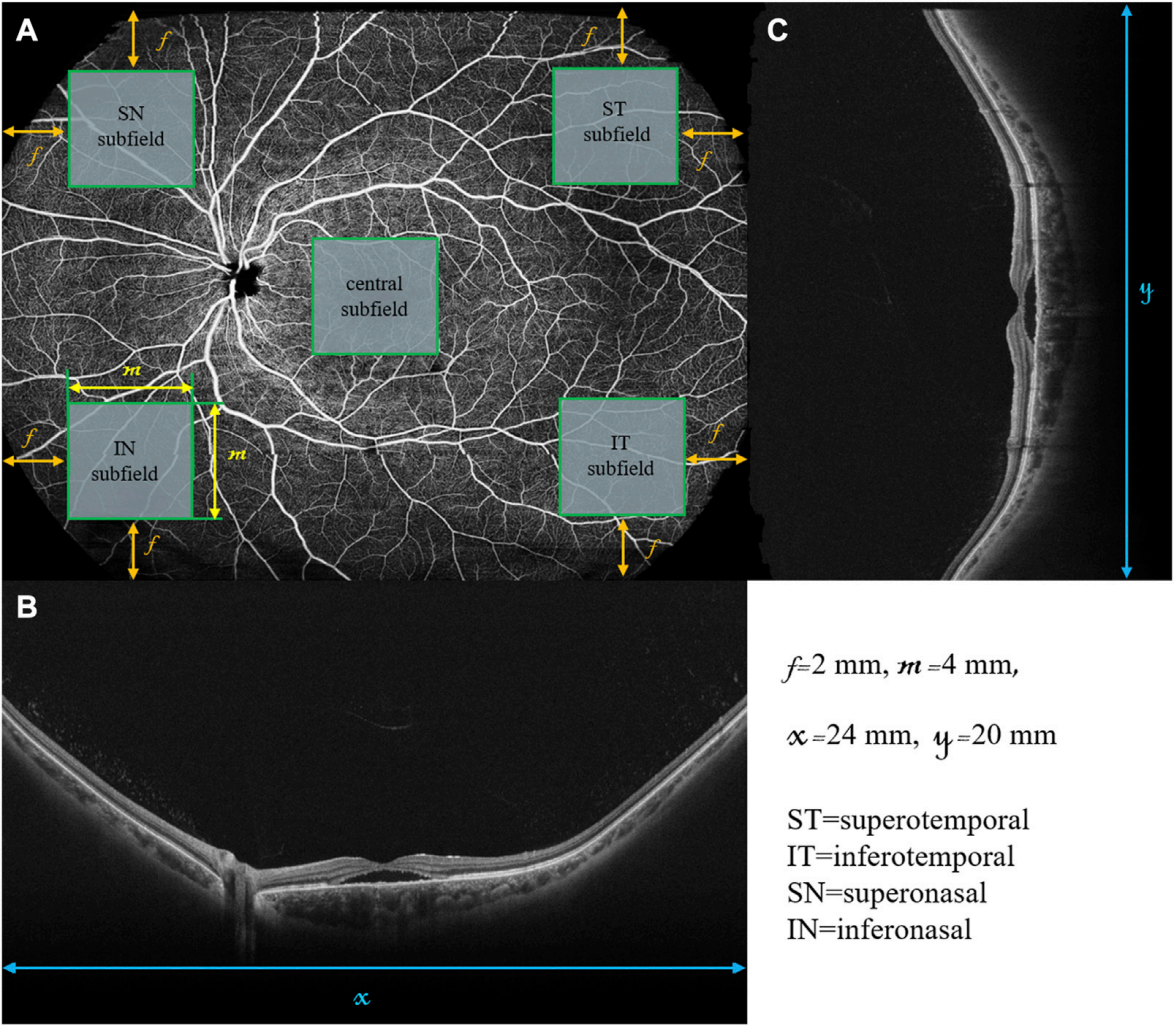
Retinal *en face* image and B scans of a diseased left eye in a patient with CSC using wide-field swept-source OCT-Angiography. The retinal *en face* image **(A)** is 24 mm in width (x) and 20 mm in height (y). The horizontal B-scan **(B)** and vertical B-scan **(C)** are 24 and 20 mm in length, respectively. The central, superotemporal (ST), inferotemporal (IT), superonasal (SN), and inferonasal (IN) subfields were represented by the five green squares, all of which were 4 by 4 mm in size (m). The central subfield is at the center of the *en face* image. The other four subfields are located in the four corners of the *en face* image with the distance between each square and the nearest edges of the *en face* image being 2 mm *(f)*.

**FIGURE 2:**
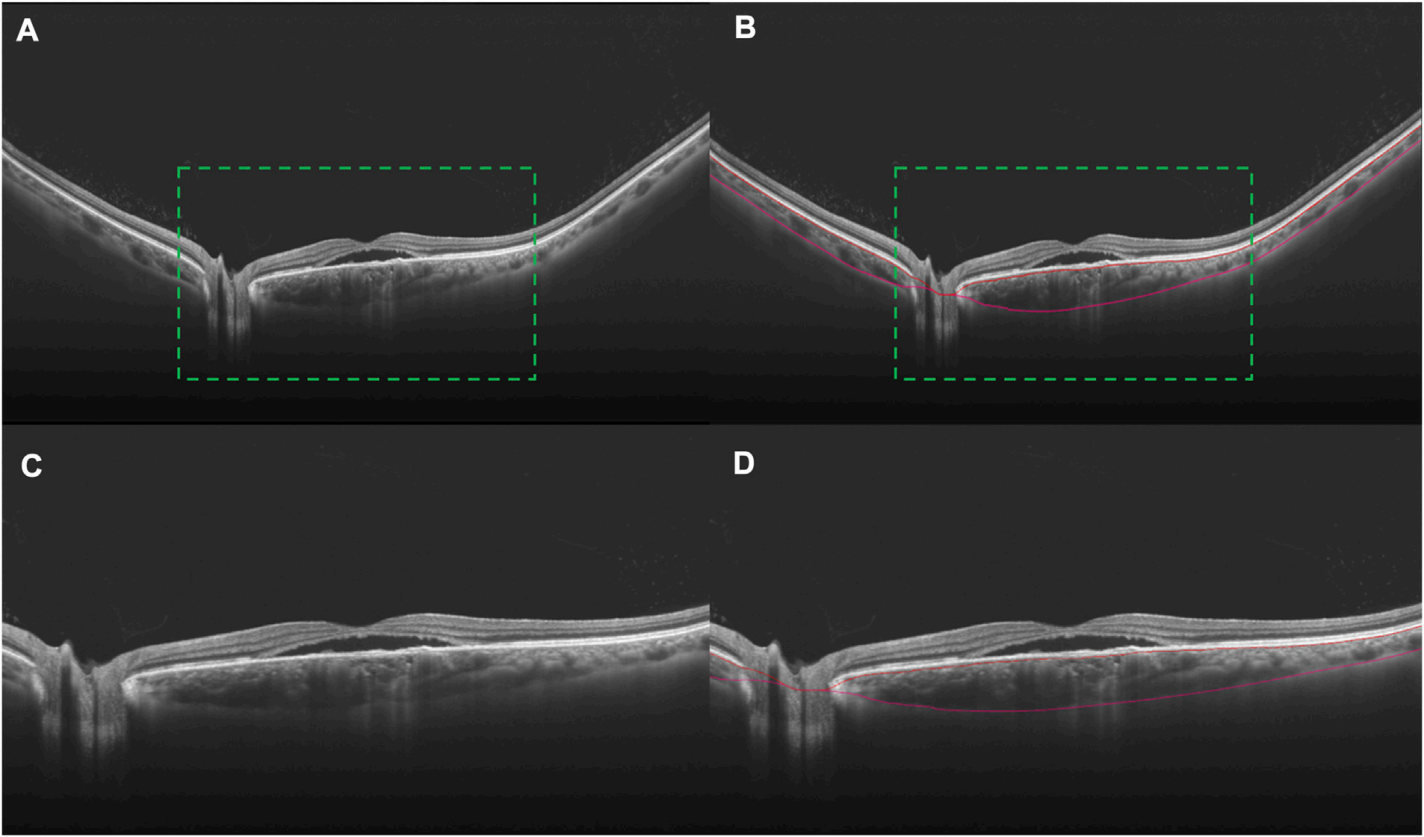
B scans indicating the segmentation of the choroid in an eye with CSC. Choroidal vasodilation can be observed, especially in the subfoveal area, in a 24 mm B scan crossing the fovea **(A)**. The choroid lies between the two marked red curves **(B)**. In order to better display the accuracy of segmentation, the images in the green dotted boxes in **(A)** and **(B)** are magnified twice to be **(C)** and **(D)**, respectively.

To assess the wide-field changes in patients with CSC, we delineated five 4-by-4 mm square regions located in the central, superotemporal, inferotemporal, superonasal, and inferonasal subfields based on the arrangement of the choroidal vasculature (Figure 1). Using wide-field ICGA, Hirahara et al. (2016) observed choroidal vasodilatation in the peripheral choroidal vessels in the eyes with CSC, especially in the areas around the vortex veins. However, few researchers have observed the peripheral areas with WF OCTA. Thus, the central subfield was centered on the fovea, representing the posterior pole while the other four subfields were located in the four quadrants of the peripheral retina as close as possible to the areas where the four vortex veins were located. For this study, choroidal thickness was defined as the distance between the Bruch’s membrane and the chorioscleral interface and the segmentation was achieved using the built-in software (Figure 2). The upper boundary of the choriocapillaris (CC) was from 29 μm posterior to the retinal pigment epithelium (RPE) while the lower boundary of CC was automatically identified and segmented.

### Statistical analysis

All data were processed and analyzed using SPSS Statistics for Windows software (Version 26.0, IBM Corporation, Armonk, NY). Variables are expressed as mean ± standard deviation (SD). Sex composition between the healthy participants and patients with CSC was analyzed using the chi-square test. For normal-distributed data, we used the *t*-tests, including both paired and unpaired *t*-tests. A paired *t*-test was used to compare differences between the diseased eyes and fellow eyes of patients with CSC while an unpaired *t*-test was used to compare differences between the healthy participants and patients with CSC. For non-normal-distributed data, we used the Wilcoxon signed-rank test and Mann-Whitney *U* test. Wilcoxon signed-rank test was used to detect differences between the diseased eyes and fellow eyes. Whereas the Mann-Whitney *U* test was used to detect differences between the healthy participants and patients with CSC.

## Results

The diseased and fellow eyes of 34 patients with unilateral CSC (29 men and 5 women), and 32 normal eyes of 32 healthy subjects (26 men and 6 women) were compared. The demographics of the participants are listed in Table 1. There was an equal representation of sexes and age between patients with CSC and the healthy subjects (*p* = 0.660 and *p* = 0.411, respectively); nor were there differences across the diseased eyes, fellow eyes, and healthy eyes and in intraocular pressure (*p* > 0.05).

**TABLE 1:** The demographic characteristics of patients with CSC and the healthy subjects.

	Patients with CSC		Healthy subjects			*p*-value
No. of subjects	34		32			
No. of eyes	68		32			
Sex (females/males)	29:5		26:6		0.660^a^	
Age (years)	48.4 ± 12.1		45.6 ± 10.8		0.411^b^	
	Diseased eyes	Fellow eyes	Healthy eyes	*p*-value^1,2^	*p*-value^2,3^	*p*-value^1,3^
IOP (mmHg)	14.4 ± 2.5	14.7 ± 2.8	15.1 ± 2.4	0.746^c^	0.416^d^	0.255^d^

^a^ The sex composition differences between the healthy participants and patients with CSC were analyzed by chi-square test.

^b^ Age differences between healthy participants and patients with CSC were analyzed by Manne-Whitney *U* test.

^c^ IOP differences between the diseased eyes and fellow eyes of patients with CSC were analyzed by paired *t*-test.

^d^ IOP differences between the eyes of patients with CSC and healthy eyes were analyzed by unpaired *t*-test.

CSC, central serous chorioretinopathy. *p-*value^1,2^, Differences between the diseased eyes and fellow eyes of patients with CSC. *p-*value^2,3^, Differences between the fellow eyes of patients with CSC and the eyes of healthy subjects. *p-*value^1,3^, Differences between the diseased eyes of patients with CSC and the eyes of healthy subjects.

WF changes in choroidal thickness among the subjects were compared (Table 2). The choroidal thickness of eyes with CSC was statistically greater than that of the fellow eyes only in the central and inferonasal subfields (*p* < 0.001 for the central subfield and *p* = 0.006 for the inferonasal subfield, respectively). The choroidal thickness of patients with CSC was statistically greater than that of healthy patients in all the subfields (*p* < 0.05 for all the five subfields in the fellow eyes and *p* < 0.05 for all the five subfields in eyes with CSC, respectively).

**TABLE 2:** Comparisons of choroidal thickness in different subfields between patients with CSC and the healthy subjects.

		Patients with CSC					
Choroidal thickness (μm)		Diseased eyes	Fellow eyes	Healthy eyes	*p-*value^1,2^	*p-*value^2,3^	*p-*value^1,3^
	Central	361.0 ± 83.2	311.8 ± 102.1	248.6 ± 54.6	<0.001	0.006	<0.001
	Superotemporal	278.9 ± 100.0	258.5 ± 73.9	186.0 ± 42.1	0.544	<0.001	<0.001
Subfields	Inferotemporal	221.2 ± 74.0	203.3 ± 76.1	166.8 ± 59.3	0.083	0.018	0.002
	Superonasal	273.4 ± 88.1	265.8 ± 100.9	233.6 ± 65.9	0.369	0.047	0.036
	Inferonasal	213.2 ± 51.1	196.8 ± 82.0	153.3 ± 39.5	0.006	0.017	<0.001

CSC, central serous chorioretinopathy. *p-*value^1,2^, Differences between the diseased eyes and fellow eyes of patients with CSC were analyzed by Wilcoxon signed-rank test. *p-*value^2,3^, Differences between the fellow eyes of patients with CSC and the eyes of healthy subjects were analyzed by Manne-Whitney *U* test. *p-*value^1,3^, Differences between the diseased eyes of patients with CSC and the eyes of healthy subjects were analyzed by Manne-Whitney *U* test.

WF changes in the vascular density of the choriocapillaris (VDcc) between subjects are summarized in Table 3. The only difference between the diseased and fellow eyes of CSC patients was in the central subfield (*p* = 0.023). The VDcc in the central and superotemporal subfields differed between the fellow eyes of CSC patients and the eyes of healthy subjects (*p* < 0.001 for the central subfield and *p* = 0.037 for the superotemporal subfield, respectively). Between the diseased eyes of CSC patients and the eyes of healthy subjects, VDcc differed in the central and superotemporal subfields (*p* = 0.021 for the central subfield and *p* = 0.018 for the superotemporal subfield, respectively).

**TABLE 3:** Comparisons of VD_CC_ in different subfields between patients with CSC and the healthy subjects.

		Patients with CSC					
VD (%)	Subfields	Diseased eyes	Fellow eyes	Healthy eyes	*p*-value^1,2^	*p*-value^2,3^	*p*-value^1,3^
	Central	55.3 ± 4.8	57.2 ± 2.7	53.9 ± 3.1	0.023	<0.001	0.021
	Superotemporal	56.6 ± 3.1	56.9 ± 3.1	58.1 ± 2.8	0.334	0.037	0.018
VD_CC_	Inferotemporal	56.7 ± 2.7	56.5 ± 2.5	56.0 ± 2.8	0.925	0.603	0.488
	Superonasal	56.7 ± 3.2	57.5 ± 3.5	56.8 ± 3.0	0.401	0.275	0.980
	Inferonasal	56.2 ± 2.9	56.7 ± 3.7	57.2 ± 2.5	0.432	0.990	0.104

VD, vascular density; CC, choriocapillaris; VD_CC_, the vascular density of the choriocapillaris; CSC, central serous chorioretinopathy. *p*-value^1,2^, Differences between the diseased eyes and fellow eyes of patients with CSC were analyzed by Wilcoxon signed-rank test. *p*-value^2,3^, Differences between the fellow eyes of patients with CSC and the eyes of healthy subjects were analyzed by Manne-Whitney *U* test. *p*-value^1,3^, Differences between the diseased eyes of patients with CSC and the eyes of healthy subjects were analyzed by Manne-Whitney *U* test.

## Discussion

Here, we quantified and compared the choroidal thickness and vascular density of the choriocapillaris of 32 normal eyes of 32 healthy subjects and 34 diseased and fellow eyes of patients with unilateral CSC using wide-field swept-source OCT-Angiography.

In recent years, OCT with enhanced depth imaging technologies has made it possible to evaluate the changes in the choroidal thickness (Daruich et al., 2015; Agrawal et al., 2020; van Rijssen et al., 2020). However, limited by the scanning area of the OCT device used, most previous studies only investigated choroidal changes in the macular region. As such, changes in the periphery regions were much less studied and it was difficult to reflect the overall situation of the choroidal changes in CSC. The OCTA we used has the largest scanning area (24 mm × 20 mm) and the highest scan rate (400,000 A-scans per second) in commercially available OCTA devices. This enabled us to measure the choroidal thickness and vascular density of CC very close to the ampulla of the vortex vein in a quick and non-invasive way. The scanning depth of 6 mm ensured that the full-thickness structure of the choroid was seen, even in patients with CSC who experienced significant choroidal thickening, pigment epithelial detachment (PED) and/or subretinal fluid. To our knowledge, there is yet no published study examing the WF choroidal changes using advanced WF SS-OCTA.

Compared with healthy subjects, diseased eyes of CSC patients had greater choroidal thickness across all subfields, suggesting that choroidal thickening existed both in the posterior pole and the periphery regions in eyes with CSC. While consistent with some prior work (Ishikura et al., 2022), this contradicts another study where eyes with CSC and healthy eyes had similar choroidal thickness in the periphery regions (Izumi et al., 2022). Here, however, the lack of difference may have resulted from their use of a single B scan to analyze choroidal thickness rather than intensive 3D scans of the entire study area (Ishikura et al., 2022; Izumi et al., 2022). We also found differences in choroidal thickness between the fellow eyes of patients with CSC and healthy eyes, indicating that some systemic factors exist in patients with CSC that affect both eyes of the patients simultaneously. Indeed, many previous studies have shown retinal and/or choroidal changes in the fellow eyes of patients with unilateral CSC (Moschos et al., 2007; Ersoz et al., 2018; Borrelli et al., 2021; Ishikura et al., 2022). Our study, together with this body of work, implied that CSC is a bilateral disease with some asymmetric manifestations (Moschos et al., 2007; Ersoz et al., 2018; Borrelli et al., 2021; Ishikura et al., 2022). It is worth noting that there were also differences in choroidal thickness between the diseased eyes and the fellow eyes, which may suggest that local factors of the diseased eyes may also play a role in the development of CSC. Using ICGA, previous studyies have showed that the delayed choroidal filling, dilated vortex veins, vortex vein anastomosis at the watershed zone, vascular congestion, and choroidal vascular hyperpermeability in the diseased eyes were all involved in the pathogenesis of CSC (Kishi et al., 2018; Matsumoto et al., 2020; Spaide et al., 2022). Whether these ICGA manifestations represent the local factors in CSC needs further exploration.

For vascular density of the choriocapillaris, the central region required extra attention. Here, VDcc was smallest in the eyes of healthy subjects and greatest in the fellow eyes of CSC patients. Interestingly, the VDcc in the diseased eyes fell in between these values. That both the diseased and fellow eyes had larger VDcc than the healthy eyes suggests that choriocapillaris was altered bilaterally in unilateral CSC. This finding also indicates that dynamic changes in CC may have happened during the development of CSC. Like in other vascular beds, both perfusion pressure and resistance determine blood flow in the choroid (Russell et al., 2022). Blood from the ophthalmic artery flows through the posterior ciliary arteries, into the Sattler’s and Haller’s layers, and then reaches the CC (Nickla and Wallman, 2010; Russell et al., 2022). From the CC, blood is drained out through the lobular venules. Those lobular venules will converge into the large choroidal veins and then the vortex veins, so that blood leaves the ocular circulation through the superior and inferior ophthalmic veins (Nickla and Wallman, 2010; Spaide 2020; Russell et al., 2022). Following a pressure gradient, the blood flows through the CC in an end-arterial manner due to the drainage through the venules (Lee et al., 2017). Choriocapillaris pressure is slightly greater than the venous pressure, which is in turn slightly greater than the IOP (Bill 1985; Mäepea 1992; Spaide et al., 2022); the pressure in the vortex vein outside of the eye is the lowest, at approximately 3–4 mm Hg (Bill 1985; Mäepea 1992; Mursch-Edlmayr et al., 2021; Spaide et al., 2022). This pressure gradient ensures that blood flows from the CC to the larger choroidal veins and then to the vortex vein outside the eye. As mentioned previously, our results suggest there may be systemic factors that affect both eyes of patients with CSC, so we assume that they are in the “pre-CSC stage”.

Several studies had demonstrated choroidal outflow congestion in eyes with CSC, so the fellow eyes could also have similar conditions (Jung et al., 2020; Jeong et al., 2022). The choroidal vascular system is similar to an electrical circuit (Spaide 2020): increasing the vascular density increases the cross-sectional area of the resistance, thereby reducing the resistance. Based on our observations, the increase in vascular density of the CC in the fellow eyes could be a compensatory response of the eye to outflow congestion. That is, it “reduces the resistance in the circuit”, so to speak, thus helping to maintain the patency of choroidal blood flow. In the diseased eyes, blood flow congestion exceeded the compensatory capacity of the choroid, disrupting the CC and resulting in a relative reduction of the vascular density.

Still, it should be pointed out that the choriocapillaris is histologically a network of capillaries that is highly anastomosed, and this complex anatomy can make the segmentation of CC challenging (Nickla and Wallman, 2010). In addition, SRF and RPE atrophy, which are commonly comorbid with CSC, also affect the CC blood flow signal detected on OCTA (Cakir et al., 2019). Besides, it should be noted that OCTA results may be variable based on external conditions and there were only a few studies on CSC using WF-OCT or OCTA, so we call for more research to strengthen or refute our findings. Why were there detectable differences in CC in some regions and not in others? We speculate that the changes in CC might be regional. Recent studies found that eyes with CSC always exhibited asymmetry in the upper and lower vortex veins and between the dominant and non-dominant sides of the vortex veins, where the dominant sides were dilated more markedly (Hiroe and Kishi, 2018; Ishikura et al., 2022). Similarly, the changes in CC might be different in different regions.

Our study did have several limitations. Our study was cross-sectional in design and included a relatively small number of patients. To further improve our understanding of the choroidal changes in CSC, future prospective and longitudinal studies are needed to monitor the choroidal changes during different stages of the disease courses. Furthermore, although the WF SS-OCTA device we used had the largest scanning area in commercially available OCTA devices, the vortex vein ampullae, which is important in the development of CSC, could not be captured simultaneously in the four quadrants. In addition, although we measured five different parts of the choroid, these regions might not be representative of the changes in the whole choroid in CSC. Besides, we included patients with a duration of symptoms from the onset of ≤9 months, yet it was challenging to accurately determine the duration of onset because some patients might not have obvious symptoms in the very early stage of the disease. And in some cases, a few patients might come to our department several months after the initial symptoms appeared. We cannot confirm whether this small number of patients have had mild and self-recovered CSC within 9 months before they came to our department. But we can be sure that they were suffering from treatment-naïve unilateral CSC at the time of their visit. Lastly, since we only excluded subjects with SE < −5D, refractive status could be a potential bias in this study and future research with emmetropic eyes would eliminate this bias. Despite these limitations, our findings using WF SS-OCTA may provide some new insight into the pathogenesis of CSC or other pachychoroid spectrum diseases.

To summarize, in this study, we found that choroidal thickening existed in the posterior pole and the periphery regions in both the diseased and fellow eyes in CSC patients compared with that found in eyes of healthy subjects. This suggests that some systemic factors affect both eyes in unilateral CSC. The diseased eyes had greater choroidal thickness than the fellow eyes, indicating that some local factors of the diseased eyes may play an important role in the development of CSC. However, further work is needed to determine what these factors are. Our results indicated that some dynamic and regional changes in vascular density occurred in the development of CSC. More broadly, we established that wide-field swept-source OCTA provides an easy, fast, non-invasive, and 3D imaging way to visualize the choroidal structure and microcirculation in the choriocapillaris in CSC. We anticipate that the rapid development of WF OCTA techniques will greatly advance our understanding of the pathogenesis of CSC.

## Data Availability

The original contributions presented in the study are included in the article/supplementary material, further inquiries can be directed to the corresponding authors.
